# Epidemiology of Current Asthma in Children Under 18: A Two-Decade Overview Using National Center for Health Statistics (NCHS) Data

**DOI:** 10.7759/cureus.49229

**Published:** 2023-11-22

**Authors:** Rhoda O Ojo, Okelue E Okobi, Patra C Ezeamii, Victor C Ezeamii, Elochukwu U Nwachukwu, Yonas H Gebeyehu, Emeka Okobi, Ademiluyi B David, Zainab Akinsola

**Affiliations:** 1 Epidemiology and Biostatistics, University of Texas Health Science Center at Houston, Houston, USA; 2 Family Medicine, Larkin Community Hospital Palm Springs Campus, Miami, USA; 3 Family Medicine, Medficient Health Systems, Laurel, USA; 4 Family Medicine, Lakeside Medical Center, Belle Glade, USA; 5 Epidemiology and Public Health, Jiann-Ping Hsu College of Public Health, Georgia Southern University, Statesboro, USA; 6 Family Medicine, University of Uyo Teaching Hospital, Uyo, NGA; 7 Medicine, Addis Ababa University, Addis Ababa, ETH; 8 Dentistry, Ahmadu Bello University Teaching Hospital, Zaria, NGA; 9 Medical Laboratory Sciences, Asokoro General Hospital, Abuja, NGA; 10 Internal Medicine/ Family Medicine, Windsor University School of Medicine, Toronto, CAN

**Keywords:** nchs-based study, epidemiology, temporal trends, children under 18, current asthma

## Abstract

Objective: This study conducted a comprehensive two-decade analysis of current asthma among children under 18 in the United States using National Center for Health Statistics (NCHS) data. The primary objective was to assess the prevalence of current asthma, evaluate temporal trends, and identify disparities based on gender, age, insurance status, household poverty levels, and race/ethnicity.

Methods: Data spanning 2003-2019 from NCHS were analyzed, focusing on current asthma prevalence among children under 18. Age-adjusted prevalence rates were calculated and stratified by various factors, including gender, age groups, health insurance status, poverty levels, and race/ethnicity.

Results: The study revealed substantial disparities in current asthma prevalence. Over the two-decade period, the overall prevalence of current asthma fluctuated. It increased from 2003 (8.5%) to 2009 (9.6%) and then decreased by 2019 (7.0%). Gender disparities were evident, with males (9.9%) consistently reporting a higher prevalence than females (7.5%). Older children aged between 10-17 years (10.4%) consistently had a higher prevalence of asthma than younger children aged 0-4 (5.3%) and 5-9 years (9.5%). Children with Medicaid insurance (11.2%) had the highest prevalence, followed by insured (8.9%), privately insured (7.7%), and uninsured children (6.1%). Children living below the federal poverty level (FPL) consistently reported the highest prevalence (11.3%), while children above 400% of the FPL (7.1%) had the lowest prevalence. Racial disparities were observed, with Black children (14.3%) having higher asthma prevalence, followed by White (7.6%) and Asian children (5.4%).

Conclusion: The study highlights significant disparities in current asthma prevalence over the two-decade period analyzed. While the overall prevalence showed fluctuations, it generally increased from 2003 to 2009 and then decreased by 2019. Gender disparities were evident, with males consistently reporting a higher prevalence compared to females. Older children in the 10-17 age group consistently had a higher asthma prevalence than younger age groups. Moreover, disparities based on insurance status and income levels were also apparent, with children on Medicaid and those living below the FPL reporting higher asthma prevalence. Racial disparities were observed, with Black children having the highest prevalence, followed by White and Asian children. These findings emphasize the importance of addressing these disparities and tailoring interventions to improve asthma management and prevention across different demographic groups.

## Introduction

Asthma is a chronic respiratory condition that affects millions of individuals worldwide, and it is one of the most common chronic diseases among children. This frequently encountered disease in society is a complex respiratory condition with multifactorial causes. Genetic predisposition plays a role, as individuals with a family history of asthma are more susceptible. Environmental factors such as exposure to airborne allergens (e.g., pollen, mold, and pet dander), pollutants (e.g., tobacco smoke, and air pollution), and respiratory infections contribute to asthma development. Early-life exposures, including viral infections during childhood, can also influence asthma risk. Additionally, occupational exposures, obesity, and certain medications may contribute to the onset or exacerbation of asthma symptoms. Understanding the diverse causes of asthma is crucial for effective prevention and management strategies tailored to individual risk factors [1-8]. 

The burden of asthma extends beyond the immediate health impact, significantly affecting the quality of life, healthcare utilization, and healthcare costs. Understanding the epidemiology of asthma in children is essential for public health planning, prevention, and effective management [1-2]. Asthma in children is of particular concern due to its prevalence, potential severity, and impact on the well-being of young individuals and their families. Asthma prevalence increased globally, affecting 262.41 million people. In the United States, over 27 million, or one in 12, suffer from asthma, with 4.5 million being children under 18 [3-4]. It is essential to monitor the epidemiological aspects of asthma to develop effective strategies for its management and prevention.

Over the past two decades, there have been notable shifts in environmental factors, healthcare practices, and socioeconomic conditions that could influence the prevalence and management of asthma among children. Additionally, ongoing efforts in public health, such as awareness campaigns and improved access to healthcare, may have impacted the epidemiology of asthma in children [5-6]. Epidemiology plays a vital role in tracking health conditions over time, identifying risk factors, and evaluating the success of interventions. By analyzing large-scale datasets, such as those provided by the National Center for Health Statistics (NCHS), epidemiologists can provide valuable insights into the patterns and determinants of current asthma in children [7]. This study provides a comprehensive overview of the epidemiology of current asthma in children under 18 years of age in the United States. By analyzing two decades of data from the NCHS, this research seeks to elucidate the trends, prevalence, and disparities in current asthma among children [8].

This research aims to comprehensively analyze current asthma in United States children under 18 over two decades. Objectives encompass assessing prevalence and temporal trends in pediatric asthma, with a focus on understanding asthma attack rates to gauge condition severity and management. Additionally, the study seeks to uncover demographic disparities in asthma, considering variables such as gender, age, insurance status, household poverty levels, and race/ethnicity. Through these objectives, the research endeavors to enhance our understanding of pediatric asthma epidemiology, including changes over time, contributing to more effective public health strategies for impacted children and their families.

## Materials and methods

Data source

This study utilized data from the NCHS, collected through the National Health and Nutrition Examination Survey (NHANES). NHANES, a cross-sectional survey conducted by the CDC, included a dataset with information on a nationally representative sample of the United States population, providing comprehensive health data, including asthma prevalence among children under 18.

Study population**

Data from the NHANES surveys conducted between 2003 and 2019 were included in the analysis, spanning a two-decade period. This timeframe allowed for a comprehensive examination of trends in current asthma prevalence among children in the United States. The study population comprised children under the age of 18 living in the United States, with a specific focus on the pediatric population vulnerable to asthma and its associated health risks.

Variables**

The primary outcome variable was the prevalence of current asthma among children under 18. Current asthma was defined as a diagnosis of asthma by a healthcare professional and the presence of asthma symptoms in the past 12 months. The analysis included demographic variables such as gender, age, race/ethnicity, insurance status, and household poverty level, enabling the examination of disparities in asthma prevalence among different population subgroups.

Statistical analysis**

Descriptive statistics were employed to calculate the overall prevalence of current asthma and to describe the study population based on demographic variables. Temporal trends in current asthma prevalence were analyzed to identify patterns and variations over the two-decade study period, involving an analysis of asthma prevalence for each year from 2003 to 2019. Subgroup analyses based on demographic variables were conducted to identify disparities in asthma prevalence. These analyses involved stratifying the data by gender, age, race/ethnicity, insurance status, and poverty level.

Ethical considerations**

This study adhered to all ethical guidelines and regulations concerning the use of de-identified health data. The data used in this analysis were anonymized, with no personally identifiable information included. Therefore, ethical approval for this secondary data analysis was not required.

## Results

The comprehensive analysis of 20 years of NCHS data provided valuable insights into the prevalence, demographic patterns, and temporal trends of current asthma among the studied population in the United States. The study revealed significant trends in the prevalence of current asthma among children under 18 in the United States from 2003 to 2019. The prevalence rate in 2003 was 8.5%, and it gradually increased to 9.6% by 2009. However, in the subsequent years, from 2010 to 2019, the prevalence showed a decreasing trend, reaching 7.0%. These findings suggest that the burden of current asthma in children under 18 experienced fluctuations during this two-decade period (Table 1)

**Table 1:** Current asthma among children under age 18, by selected characteristics * * Data not available

Characteristic, Percentage (Standard error)		2003	2004	2005	2006	2007	2008	2009	2010	2011	2012	2013	2014	2015	2016	2017	2018	2019
	Total, under 18 years	8.5 (0.3)	8.5 (0.3)	8.9 (0.3)	9.3 (0.4)	9.1 (0.4)	9.4 (0.4)	9.6 (0.4)	9.4 (0.3)	9.5 (0.3)	9.3 (0.3)	8.3 (0.3)	8.6 (0.3)	8.4 (0.3)	8.3 (0.3)	8.4 (0.4)	7.5 (0.4)	7 (0.3)
Age group	0–4 years	5.9 (0.5)	5.6 (0.4)	6.8 (0.5)	5.8 (0.6)	6.8 (0.6)	6.2 (0.6)	6.3 (0.6)	6 (0.5)	6.9 (0.5)	5.4 (0.5)	4.2 (0.4)	4.3 (0.5)	4.7 (0.5)	3.8 (0.5)	4.4 (0.6)	3.8 (0.5)	2.6 (0.4)
	5–9 years	9 (0.6)	8.7 (0.6)	9.7 (0.6)	11.5 (0.8)	9.2 (0.8)	11.1 (0.8)	10.2 (0.7)	10.6 (0.7)	9.7 (0.6)	10.6 (0.6)	9 (0.6)	10.2 (0.7)	9 (0.6)	9.2 (0.7)	8.4 (0.7)	7.4 (0.7)	7.8 (0.7)
	10–17 years	9.8 (0.5)	10.1 (0.5)	9.8 (0.5)	10.2 (0.6)	10.5 (0.7)	10.5 (0.6)	11.4 (0.6)	10.8 (0.5)	11.1 (0.5)	10.8 (0.5)	10.4 (0.5)	10.2 (0.6)	10.3 (0.5)	10.5 (0.6)	10.8 (0.6)	9.9 (0.6)	9.1 (0.5)
Sex	Male	9.5 (0.4)	10.2 (0.4)	10 (0.4)	11 (0.6)	9.7 (0.5)	11.4 (0.6)	11.3 (0.6)	10.5 (0.5)	10.2 (0.5)	10 (0.5)	9.3 (0.4)	10.1 (0.5)	9.9 (0.5)	9.2 (0.5)	9.5 (0.6)	8.3 (0.5)	8.4 (0.5)
	Female	7.5 (0.4)	6.7 (0.4)	7.8 (0.4)	7.5 (0.5)	8.5 (0.6)	7.4 (0.5)	7.9 (0.5)	8.2 (0.4)	8.8 (0.5)	8.6 (0.4)	7.3 (0.4)	7 (0.5)	6.9 (0.4)	7.4 (0.5)	7.3 (0.5)	6.7 (0.5)	5.5 (0.4)
Race	White only	7.4 (0.3)	7.8 (0.3)	8 (0.3)	8.7 (0.4)	7.7 (0.4)	8.2 (0.4)	8.2 (0.4)	8.1 (0.4)	8 (0.4)	8 (0.4)	7.2 (0.3)	7.9 (0.4)	7.5 (0.4)	6.8 (0.4)	7.6 (0.4)	6.1 (0.4)	5.9 (0.4)
	Black or African American only	13.2 (0.9)	12.5 (0.9)	13.2 (0.9)	12.8 (0.9)	15.6 (1.1)	15.5 (1.1)	16.8 (1.2)	15.8 (1)	16.4 (1.1)	15.8 (1)	13.8 (0.9)	13 (1)	13.5 (1)	15.1 (1.2)	12.8 (1.2)	14.1 (1.4)	13.5 (1.3)
	American Indian or Alaska Native only	16.2 (4)	* *	* *	* *	* *	16.4 (4.8)	* *	* *	* *	14.5 (3.6)	* *	10.2 (2.5)	11.4 (2.8)	15.1 (4.4)	* *	* *	* *
	Asian only	* *	3.4 (0.9)	6.5 (1.3)	6.3 (1.4)	7.4 (1.5)	3.7 (1)	7.7 (1.4)	8.4 (1.4)	7 (1)	5.1 (1)	4.9 (1)	5.3 (1.1)	5.3 (1)	4 (0.9)	3.8 (0.8)	3.9 (0.9)	4.2 (1)
Percent of poverty level	Below 100%	10.9 (0.8)	9.6 (0.8)	10.6 (0.8	12.2 (0.9	11.4 (1)	11.5 (1.1)	13.5 (1.1)	12.1 (0.9)	12.5 (0.8)	13 (0.8)	11.7 (0.8)	10.5 (0.7)	10.7 (0.8)	10.5 (0.9)	11 (1.1)	10.2 (1.2)	10.5 (1.1)
	100%–199%	8.3 (0.7)	9.3 (0.7)	8.3 (0.7	9.6 (0.8	9.8 (1)	10.2 (0.8)	9.4 (0.8)	10.2 (0.7)	10.2 (0.7)	9.3 (0.6)	8.1 (0.6)	7.9 (0.6)	9.4 (0.8)	9.9 (0.8)	9.1 (0.8)	9 (0.9)	7.2 (0.7)
	200%–399%	8.4 (0.6)	7.6 (0.5)	9 (0.6	9.2 (0.7	8.4 (0.6)	8.7 (0.7)	7.3 (0.6)	8.6 (0.6)	9 (0.6)	8.5 (0.6)	7.6 (0.6)	8.8 (0.7)	7.4 (0.6)	7.4 (0.6)	8.1 (0.6)	7 (0.6)	6.8 (0.5)
	400% or more	7.4 (0.6)	8.1 (0.6)	8.2 (0.5	6.9 (0.6	7.8 (0.7)	8.2 (0.7)	9.3 (0.7)	7.2 (0.5)	6.8 (0.5)	6.9 (0.5)	6.4 (0.6)	7.4 (0.6)	7 (0.6)	6.6 (0.5)	6.7 (0.5)	5.5 (0.5)	4.9 (0.5)
Health insurance status	Insured	8.8 (0.3)	8.9 (0.3)	9.2 (0.3	9.7 (0.4	9.5 (0.4)	9.8 (0.4)	9.9 (0.4)	9.6 (0.3)	9.7 (0.3)	9.5 (0.3)	8.4 (0.3)	8.7 (0.4)	8.5 (0.3)	8.4 (0.3)	8.6 (0.4)	7.7 (0.4)	7.2 (0.3)
	Private	7.6 (0.4)	8.4 (0.4)	8.1 (0.4	8.3 (0.5	8.1 (0.5)	8.6 (0.5)	8.5 (0.5)	8.1 (0.4)	7.9 (0.4)	8.1 (0.4)	7 (0.4)	8 (0.5)	7.3 (0.4)	7.2 (0.4)	7.5 (0.4)	6.8 (0.5)	5.2 (0.4)
	Medicaid	11.9 (0.7)	10.5 (0.7)	11.9 (0.7	12.8 (0.8	12.6 (0.9)	12 (0.8)	11.9 (0.7)	12.1 (0.6)	12.2 (0.6)	11.6 (0.6)	10.4 (0.5)	9.8 (0.5)	10.3 (0.6)	10.1 (0.6)	10.8 (0.7)	9.4 (0.7)	10.2 (0.7)
	Uninsured	6.1 (0.8)	4.1 (0.6)	6.5 (1	5.8 (0.8	5.6 (0.8)	6.4 (1.1)	7 (1)	6.4 (1)	7 (1.1)	6.6 (1)	7.1 (1.1)	6.9 (1.2)	6.8 (1.4)	8.3 (1.7)	5.6 (1.3)	5.1 (1.2)	2.5 (0.8)

Based on gender

**Figure 1:**
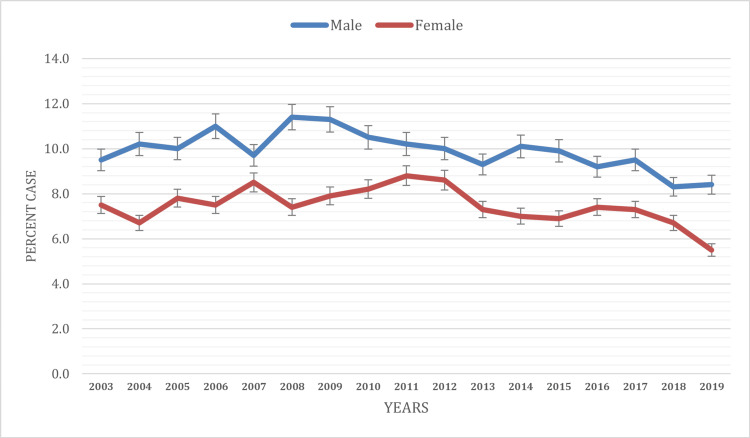
Current asthma prevalence based on gender

Gender disparities in current asthma prevalence were evident throughout the study period. Males (9.9%) consistently displayed a higher prevalence of current asthma than females (7.5%) during the study period. In 2003, the prevalence of current asthma among males was 9.5%, while among females, it was 7.5%. Over the following years, these prevalence rates showed distinctive patterns. By 2009, the prevalence of current asthma had increased among both males (11.3%) and females (7.9%). This increasing trend continued until 2011 (8.8%) in females, while male prevalence reached its peak at 11.3% in 2009 only and showed a decreasing trend after that. Gender-based prevalence trends showed variations. The prevalence of current asthma among males exhibited a declining pattern, reaching 8.4% by 2019, and 5.5% in females (Figure 1).

Based on the age of the children

The results emphasize the age-specific disparities in current asthma prevalence among children under 18. While younger children aged 0-4 (5.3%) and 5-9 years (9.5%) reported lower prevalence rates, children aged 10-17 years (10.4%) consistently reported the highest prevalence of current asthma during the study period. In 2003, the prevalence of current asthma was 5.9% among children aged 0-4 years, gradually increasing to 6.9% by 2011.

**Figure 2:**
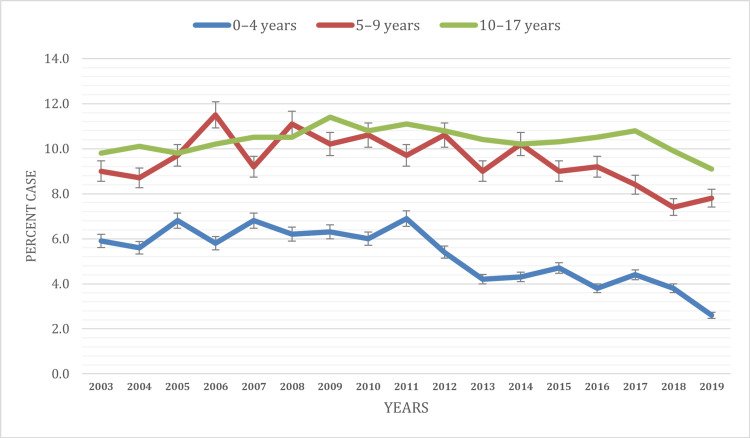
Current asthma prevalence based on the age of the children

Similarly, children aged 5-9 years experienced an increase in prevalence, rising from 9.0% in 2003 to 11.5% in 2006. From 2011 to 2019, the prevalence of current asthma among children aged 0-4 continued to show a declining trend. However, children aged 5-9 years exhibited fluctuating trends from 2007 (9.2%) to 2019 (7.8%). In contrast, the age group of 10-17 years saw consistent asthma prevalence during the entire study period. In 2003, 9.8% of children in this age group had current asthma, which steadily rose to 9.9% by 2018 and decreased to 9.1% in 2019 (Figure 2).

Based on race

This study aimed to explore the race-specific prevalence of current asthma among children under 18 in the United States from 2003 to 2019, providing insights into disparities in asthma rates among different racial groups. In 2003, the prevalence of current asthma varied significantly among racial groups. The highest prevalence was observed among American Indian or Alaska Native children (16.3%), followed by Black children (13.2%). White children had a lower prevalence of 7.4%, while data for Asian children were not available for 2003 (Table 1).

**Figure 3:**
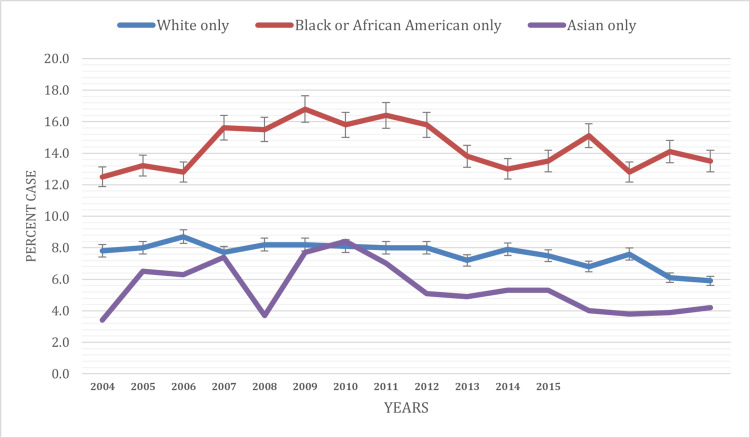
Current asthma prevalence based on race

Over the study period, current asthma prevalence exhibited varying trends among different racial groups. Black children experienced fluctuations in prevalence, with rates reaching 16.8% by 2009, dropping to 13.0% in 2014, and then increasing to 13.5% by 2019. In White children, there was a declining trend, with prevalence rates falling from 7.4% in 2003 to 5.9% in 2019. Among Asian children, the prevalence showed variations, with rates reaching 8.4% by 2010 from 3.4% in 2004, dropping to 4.2% in 2019. Data for American Indian or Alaska Native children were reported for a few random years only, making trend analysis difficult (Figure 3).

Based on the percentage of the poverty level

This study aimed to examine the prevalence of current asthma among children under 18 in the United States from 2003 to 2019, focusing on disparities based on household poverty levels.

In 2003, the prevalence of current asthma varied among children from different poverty levels. Children living below the federal poverty level (FPL) reported the highest prevalence at 10.9%. Those living between 200-399% of the FPL had a slightly lower prevalence of 8.3%, followed by 100-199% of FPL with 8.3%, while children living above 400% of the FPL exhibited the lowest prevalence at 7.4%.

Over the two-decade study period, trends in current asthma prevalence based on poverty levels displayed fluctuations and disparities. Children living below the FPL saw their prevalence rates rise from 10.9% in 2003 to 13.5% in 2009, followed by a gradual decline to 10.5% in 2019.

**Figure 4:**
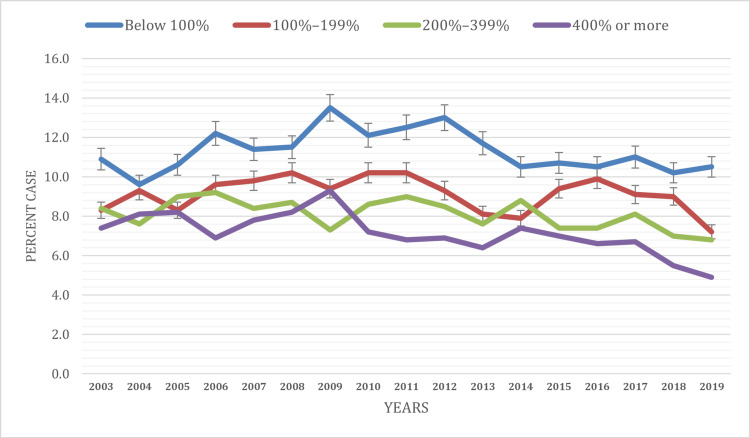
Current asthma prevalence based on the percentage of the poverty level Below 100%: Individuals or families in this category have incomes that are less than the federal poverty level; 100%–199%: Individuals or families in this category have incomes that are between 100% and 199% of the federal poverty level; 200%–399%: Individuals or families in this category have incomes that are between 200% and 399% of the federal poverty level; 400% or more: Individuals or families in this category have incomes that are 400% or more of the federal poverty level.

For children in the 100-199% FPL group, asthma prevalence exhibited an increasing pattern, with rates increasing from 8.3% in 2003 to 9.1% in 2017, then decreasing to 7.2% in 2019. Children living between 200-399% of the FPL saw their prevalence rates rise from 8.4% in 2003 to 9.0% in 2011, followed by a gradual decline to 6.8% in 2019. Children living above 400% of the FPL reported a declining trend in asthma prevalence, decreasing from 7.4% in 2003 to 4.9% in 2019 (Figure 4). These findings underscore the existence of poverty-level-based disparities in current asthma prevalence among children under 18 in the United States. Notably, children from families living below the FPL consistently reported the highest prevalence of asthma, which exhibited significant fluctuations. Children from families with incomes between 100-199% of the FPL experienced relatively stable asthma prevalence rates, while those living above 200% of the FPL displayed declining trends over the study period.

Based on health insurance status 

The findings highlight health insurance status-based disparities in current asthma prevalence among children under 18 in the United States. Children with Medicaid insurance reported the highest prevalence at 11.2%, followed by those who were insured (8.9%), had private health insurance (7.7%), and those who were uninsured (6.1%) during the study period.

In 2003, the prevalence of current asthma showed variations among children based on their health insurance status. Children with Medicaid health insurance reported a prevalence rate of 11.9%, insured children reported 8.8%, those with private health insurance had a slightly lower prevalence of 7.6%, and uninsured children exhibited the lowest prevalence at 6.1%.

**Figure 5:**
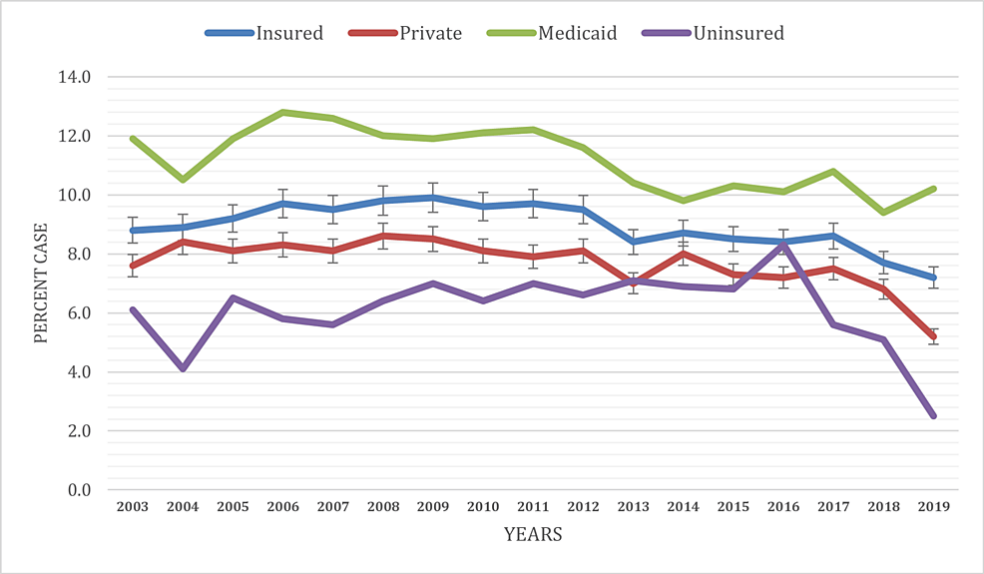
Current asthma prevalence based on health insurance status at the time of the interview

Over the two-decade study period, the trends in current asthma prevalence based on health insurance status displayed varying patterns. Children with private health insurance saw a decline in asthma prevalence from 7.6% in 2003 to 5.2% in 2019, indicating consistent improvement in their health outcomes. Children with Medicaid insurance also experienced a declining trend in asthma prevalence, decreasing from 11.9% in 2003 to 10.2% in 2019. Uninsured children, on the other hand, exhibited fluctuations in asthma prevalence, with rates peaking at 7.0% in 2011 and then gradually declining to 2.5% in 2019 (Figure 5).

## Discussion

The study examined trends in asthma prevalence and identified significant disparities based on gender, age, insurance status, household poverty levels, and race/ethnicity. The disparities in asthma prevalence based on sociodemographic variables underscore the importance of tailored public health interventions. Addressing these disparities is critical for reducing the overall burden of pediatric asthma.

The study revealed consistent gender disparities in current asthma prevalence among children. Males consistently displayed a higher prevalence of current asthma than females throughout the two-decade period. These findings align with previous research and consistently reported similar gender disparities [1, 9-10]. Potential reasons for this difference could include hormonal influences, genetic factors, and varying asthma triggers to which boys and girls may be exposed. However, more research is needed to better understand these disparities, as they have important implications for public health initiatives.

The age-specific patterns in asthma prevalence indicate that children aged 10-17 consistently reported higher asthma prevalence than younger children aged 0-9. This finding is in line with Pakkasela et al., which noted that asthma severity and prevalence often increase with age [11-12]. Recognizing these age-specific patterns is crucial for tailoring healthcare and prevention strategies to different age groups.

The study identified significant racial and ethnic disparities in current asthma prevalence among children, with Black children, in particular, experiencing notable fluctuations. Similar disparities have been reported in a study conducted by Children’s Respiratory and Environmental Workgroup [13-15]. These disparities are likely multifactorial, with factors such as socioeconomic status, housing conditions, and environmental exposures playing critical roles. Recognizing these disparities is vital for developing targeted interventions and healthcare policies that address the specific needs of affected racial and ethnic groups.

Children living below the FPL consistently reported higher asthma prevalence, mirroring the findings of Sullivan et al. [16]. Poverty often leads to suboptimal housing conditions, limited access to healthcare, and increased exposure to environmental triggers, all factors contributing to higher asthma prevalence [17-18].

Children with different insurance statuses displayed varying rates of current asthma. Those with Medicaid insurance consistently reported the highest prevalence. These findings are consistent with studies such as Gushue et al., which have noted the impact of insurance status on pediatric asthma outcomes [19]. Children with Medicaid insurance may face disparities in healthcare access, potentially leading to higher prevalence rates. Conversely, privately insured children demonstrated a declining trend in asthma prevalence, indicating improved healthcare access. [20] These findings underscore the importance of health insurance policies and their impact on pediatric asthma outcomes. Healthcare policies should aim to improve healthcare access and quality for vulnerable groups. Additionally, public health campaigns should focus on asthma prevention and management, with an emphasis on reaching communities facing the highest disparities.

The study also poses several limitations. Utilizing data from NCHS in research offers valuable insights, but it may come with inherent limitations that researchers should consider. One notable limitation is the potential for sampling bias, as NCHS data often relies on specific surveys and sampling methods that may not fully capture the diversity of the entire population. Additionally, the accuracy of the data is contingent on the reliability of reporting and documentation, introducing the possibility of errors that can impact the precision of findings. These temporal limitations may pose challenges when studying rapidly changing health trends or events between data collection intervals. So, while NCHS data provides a broad overview, there may be challenges in generalizing findings to specific subpopulations or smaller geographic areas. Despite these known limitations, recognizing the strengths and weaknesses of NCHS data is crucial to making informed decisions and employing complementary data sources or methodological approaches to enhance the robustness of their research. A significant limitation is the absence of data on Asian children for the early years of the study period, limiting the ability to assess trends within this population. The dataset also lacks comprehensive information on non-medical determinants of asthma, such as environmental factors and lifestyle, which are essential in understanding the full spectrum of asthma risk and prevalence. The study's cross-sectional nature makes it challenging to establish causality between variables and asthma prevalence.

This study presents several notable strengths. Firstly, it conducts a comprehensive analysis of current asthma in children under 18 over a substantial two-decade period, providing valuable insights into the long-term trends and patterns. The utilization of the NCHS data is a major strength, as it ensures a large and nationally representative dataset. This robust dataset allows for reliable and generalizable findings, enhancing the study's credibility. Additionally, the study's stratified approach to analyzing asthma prevalence by gender, age, insurance status, poverty levels, and race/ethnicity adds depth to the analysis, providing a nuanced understanding of disparities.

Future research should focus on the underlying factors contributing to these disparities, including a more nuanced analysis of environmental exposures, healthcare access, and genetic influences. Longitudinal studies can help track changes in prevalence and identify trends associated with interventions and policy changes. Comparative research across regions and countries can further elucidate the universal and unique aspects of asthma epidemiology.

## Conclusions

In conclusion, the study highlights significant disparities in current asthma prevalence over the two-decade period analyzed. While the overall prevalence showed fluctuations, it generally increased from 2003 to 2009 and then decreased by 2019. Gender disparities were evident, with males consistently reporting a higher prevalence compared to females. Older children in the 10-17 age group consistently had a higher asthma prevalence than younger age groups. Moreover, disparities based on insurance status and income levels were also apparent, with children on Medicaid and those living below the FPL reporting higher asthma prevalence. Racial disparities were observed, with Black children having the highest prevalence, followed by White and Asian children. Recognizing and addressing disparities in asthma prevalence is vital for achieving equitable health outcomes, tailoring targeted interventions, informing public health planning, and reducing overall health inequalities.
